# Financialization, Food Sovereignty, and Oral Health: A Structured Narrative Review Within the One Health Framework for Sustainable Food Systems

**DOI:** 10.3390/foods15152718

**Published:** 2026-08-01

**Authors:** Maria Antoniadou, Theodoros Varzakas, Martin Caraher

**Affiliations:** 1 Department of Dentistry, School of Health Sciences, National and Kapodistrian University of Athens, 11527 Athens, Greece; mantonia@dent.uoa.gr; 2 Department Food Science and Technology, University of the Peloponnese, 24100 Kalamata, Greece; 3 Centre for Food Policy, City St. George’s, University of London, London EC1R 1UW, UK; m.caraher@citystgeorges.ac.uk

**Keywords:** food security, food sustainability, food sovereignty, food-system financialization, One Health, oral health, oral microbiome, ultra-processed foods, food environments, food-system performance

## Abstract

Contemporary food systems are increasingly shaped by financialization, corporate concentration, and unequal distributions of power that influence food production, food environments, dietary exposures, and population health. However, the relationships among food-system financialization, food sovereignty, and oral health remain insufficiently integrated within food-security, sustainability, and One Health research. This structured narrative review critically synthesized interdisciplinary evidence from Scopus, Web of Science, PubMed/MEDLINE, Google Scholar, citation searching, and authoritative institutional sources to examine these relationships and develop an integrative conceptual framework. The synthesis indicates that financialization may influence health through market concentration, commodity dependence, corporate control of food environments, and the expansion of ultra-processed foods, whereas food sovereignty may modify these pathways by strengthening agency, equitable resource distribution, local governance, and ecological resilience. Dietary exposures and related biological mechanisms provide plausible pathways through which these structural processes may contribute to oral-health outcomes and inequalities. The proposed framework integrates food-system structures, governance, food environments, dietary exposures, biological pathways, oral health, and One Health implications within a common analytical model. Oral health is therefore proposed as a potential biological interface through which food-system transformations and inequalities may become measurable. Empirical research is required to test these pathways and evaluate the framework’s applicability across populations and food-system contexts.

## 1. Introduction

Contemporary food systems are undergoing profound structural transformations driven by industrialized agricultural production, globalization, technological innovation, corporate consolidation, climate change, and changing patterns of food governance [1,2]. Although these developments have increased agricultural productivity and expanded global food supply, they have also contributed to monoculture production, biodiversity loss, environmental degradation, market concentration, and increasing vulnerability to economic, geopolitical, and ecological disruptions [3,4]. These tensions reflect competing conceptions of food as a market commodity and as a social and ecological commons, with important implications for food-system governance, equity, and sustainability [1].

Recent crises, including climate-related disruptions, armed conflicts, pandemics, inflationary pressures, and changing international trade relations, have exposed persistent inequalities in food production, distribution, and access [2,3]. The consequences are disproportionately experienced by low- and middle-income countries, small-scale producers, and socioeconomically disadvantaged populations, demonstrating that food insecurity cannot be understood solely as a problem of insufficient production or aggregate food availability [2,3,4]. Sustainable agri-food transformation therefore requires food systems capable of providing safe, nutritious, and high-quality food while simultaneously supporting environmental sustainability, economic viability, social justice, and resilience [5,6]. However, prevailing development trajectories continue to favor technological intensification, increasing farm size, corporate concentration, and large-scale agribusiness, generating tensions between productivity, environmental objectives, producer livelihoods, and equitable access to productive resources [7,8,9,10,11]. The social and economic organization of agriculture further illustrates these inequalities. Despite its central contribution to employment and livelihoods, particularly in low- and middle-income countries, agriculture generates a comparatively small share of global economic value, while smallholder farmers frequently operate with limited market power, restricted access to productive resources, and disproportionate exposure to economic and environmental risks [12,13,14]. These asymmetries reinforce the need to evaluate food systems beyond aggregate productivity, trade efficiency, and food availability [13,14].

Against this background, the financialization of food systems has emerged as a significant structural transformation affecting contemporary agriculture and food governance [15,16]. Financialization refers to the increasing influence of financial markets, institutions, investment strategies, and shareholder-oriented business models on agricultural production, commodity trading, land ownership, agribusiness, and food-system decision-making [15,16,17,18,19]. Commodity speculation, financial investment in agricultural land and businesses, commodity index funds, and corporate mergers and acquisitions have expanded the role of financial actors whose objectives may be increasingly disconnected from the physical production and distribution of food [15,16,17]. Although commodity markets can perform legitimate functions in price discovery and risk management, the growing influence of speculative investment and concentrated market power has raised concerns regarding food-price volatility, producer vulnerability, transparency, accountability, and the unequal distribution of economic value throughout food supply chains [18,19,20]. Financialization is therefore relevant not only to agricultural markets but also to the distribution of power and resources across food systems [15,16]. It may influence food production and processing, food environments, and population dietary exposures by shaping investment priorities, ownership, corporate strategies, and market concentration, although the pathways linking these upstream structures with measurable biological outcomes remain insufficiently integrated within food-system research [15,16,19]. These processes may consequently shape food environments, supply chains, population dietary exposures, social inequalities, and health outcomes, although the pathways connecting financial structures with measurable biological consequences remain insufficiently integrated within food-system research [15,16].

The concentration of economic and decision-making power associated with financialized food systems is closely related to debates concerning food sovereignty [21]. Food sovereignty shifts attention from aggregate food availability toward the rights of people and communities to participate in decisions concerning food production, distribution, and consumption and to maintain greater control over productive resources and food-system governance [22]. The concept emphasizes democratic participation, producer and community agency, culturally appropriate food, ecological sustainability, equitable livelihoods, and the redistribution of power within food systems [21,22]. Financialization and food sovereignty therefore provide complementary analytical perspectives: the former examines how financial actors and mechanisms influence ownership, investment, markets, and value extraction, whereas the latter addresses who retains decision-making power, whose interests are represented, and whether producers and communities possess meaningful agency over food systems [15,16,21,22].

These structural determinants are increasingly relevant to contemporary food-security frameworks, which have expanded beyond availability, access, utilization, and stability to recognize agency and sustainability as essential conditions for equitable and resilient food systems [23]. This broader perspective acknowledges that dietary exposures are shaped by social, commercial, economic, and political processes governing food production, processing, pricing, marketing, and access [24,25,26,27,28,29,30,31,32,33]. Within increasingly concentrated food systems, the global expansion of ultra-processed foods illustrates how industrial scalability, market power, and corporate strategies can reshape food environments and population diets, increasing exposure to dietary patterns associated with noncommunicable diseases and adverse oral-health outcomes [34,35,36,37,38,39,40,41,42,43].

Despite growing attention to food systems, sustainability, and health equity, oral health remains largely absent from food-security and food-system performance frameworks. Yet, the oral cavity represents the first biological interface with dietary exposures, where food composition, processing, sugar content, acidity, and consumption frequency influence microbial ecology and oral disease development [44,45,46,47]. Oral-health outcomes may therefore provide measurable evidence of the biological embodiment of socially patterned dietary exposures [48,49,50,51].

The One Health perspective provides a systems-based foundation for integrating these relationships by recognizing the interdependence of human health, food production, environmental conditions, and microbial ecosystems [47,52,53]. Within this systems-based perspective, incorporating oral health may extend existing frameworks by connecting upstream food-system organization and dietary exposures with downstream biological outcomes, health inequalities, and population well-being [45,46,47].

Accordingly, this review addresses the limited integration of food-system financialization, food sovereignty and governance, and the pathways linking food environments and dietary exposures with oral-health outcomes [15,16,23,44,45,46,47,54,55]. Financialization of food systems has not been studied in food policy analysis. Likewise food-system and One Health frameworks rarely incorporate oral health as a potential biological measure of structurally patterned dietary exposures and inequalities [54,55]. This structured narrative review therefore critically synthesizes these interdisciplinary domains and develops an integrative One Health framework linking food-system organization, governance, dietary exposures, and oral-health outcomes. Specifically, the review addresses four questions: (RQ1) How does food-system financialization impact food production, market concentration, food environments and population dietary exposures? (RQ2) How does food sovereignty shape agency, equity, resilience, and sustainability within contemporary food systems? (RQ3) Through which dietary, ecological, and biological pathways do food-system transformations influence oral-health outcomes? (RQ4) How can oral health be incorporated into a One Health framework as a potential indicator of food-system sustainability, equity, and resilience?

## 2. Materials and Methods

### 2.1. Review Design and Scope

This study was conducted as a structured narrative review with conceptual framework development. A narrative approach was selected because the review question spans heterogeneous and interdisciplinary bodies of literature, including the food-system political economy, financialization, food sovereignty, food security, sustainability, food environments, nutrition, oral health, and One Health. The purpose was therefore not to estimate pooled effects or provide an exhaustive systematic inventory of studies, but to critically integrate theoretical, empirical, and policy-oriented evidence and identify relationships among structural food-system drivers, dietary exposures, biological pathways, and oral-health outcomes. The review was guided by four predefined questions concerning (1) the influence of financialization on food production, market concentration, food environments, and dietary exposures; (2) the role of food sovereignty in agency, equity, resilience, and sustainable food-system governance; (3) the dietary, ecological, and biological pathways linking food-system transformations with oral-health outcomes; and (4) the potential incorporation of oral health into a One Health framework for assessing food-system sustainability, equity, and resilience.

### 2.2. Information Sources and Literature Search

A structured and iterative literature search was conducted to identify publications relevant to the predefined review questions. The literature was retrieved from Scopus, Web of Science Core Collection, PubMed/MEDLINE, and Google Scholar. Searches were supplemented by backward citation searching of the reference lists of relevant publications and forward citation searching to identify subsequent studies that extended or critically discussed key concepts and findings. The search strategy combined terms related to four main conceptual domains: (1) food-system structure and political economy, including food system, financialization, commodity markets, food trade, corporate concentration, agribusiness, and land acquisition; (2) food sovereignty and sustainable governance, including food sovereignty, food security, agency, food-system governance, agroecology, smallholder farmers, equity, resilience, and sustainability; (3) food environments and dietary exposures, including food environments, dietary patterns, nutrition transition, ultra-processed foods, food processing, free sugars, and dietary inequalities; and (4) oral health and One Health, including oral health, dental caries, dental erosion, periodontal disease, oral microbiome, saliva, mastication, oral-health inequalities, and One Health. Search terms were combined using Boolean operators and adapted to the syntax and indexing requirements of each database.

The search focused primarily on peer-reviewed publications in English, while authoritative reports and policy documents from international organizations were considered when necessary to establish major food-security, sustainability, and governance frameworks. Once key authors were identified we searched on their websites to identify grey literature (government and NGO reports) and other publications. This later group of publications was used in the discussion but not included in the literature selection to inform the findings.

No restrictions were imposed regarding geographical setting because the review examined global food-system processes and their implications across different socioeconomic and policy contexts. The literature search covered publications published between 2008 and May 2026. The year 2008 was selected as the starting point because it marked an important turning point in the evolution of contemporary food systems following the Great Recession and coincided with the emergence of an expanding body of interdisciplinary literature relevant to the review objectives.

### 2.3. Eligibility and Literature Selection

Publications were considered eligible when they contributed directly to at least one of the predefined review domains and provided relevant theoretical, empirical, mechanistic, or policy evidence. Eligible sources included original research articles, systematic and narrative reviews, conceptual and theoretical studies, and authoritative institutional reports addressing food-system financialization, commodity markets, corporate concentration, agricultural restructuring, food sovereignty, food security, sustainability, food environments, dietary exposures, ultra-processed foods, oral-health outcomes, or One Health-related interactions. Publications were excluded when they addressed these concepts only tangentially, lacked a clear relationship with the review questions, duplicated evidence already represented by more comprehensive or recent sources, or did not contribute to the interpretation or development of the proposed framework. Editorials, commentaries, and non-peer-reviewed materials were generally excluded unless they represented influential conceptual contributions or authoritative policy sources necessary for interpreting the historical or institutional development of the field. Literature selection followed an iterative process based on conceptual relevance, methodological contribution, and scientific credibility. Citation searching was used to identify additional sources that clarified emerging themes and relationships. Selection continued until thematic saturation was reached, defined as the point at which additional sources no longer substantially modified the principal themes or conceptual relationships identified in the review.

### 2.4. Data Extraction and Thematic Synthesis

Relevant information was extracted and organized according to the predefined review questions and analytical domains. Evidence was synthesized using a combined deductive and inductive thematic approach, allowing both predefined concepts and additional cross-cutting relationships to emerge from the literature. The synthesis focused on identifying relationships across structural drivers, food-system processes, dietary exposures, biological pathways, and oral-health outcomes. Areas of convergence, divergence, and evidence gaps were examined to support the integration of findings and the subsequent development of the conceptual framework. The core sources that informed the thematic synthesis and development of the conceptual framework are summarized in Table 1. These publications were selected for their direct contribution to the principal analytical domains of the review and to the identification of relationships among structural food-system drivers, governance arrangements, dietary exposures, biological pathways, oral-health outcomes, and One Health implications. Together, these sources provided the theoretical, empirical, and framework-level evidence used to organize the narrative synthesis and develop the proposed integrative model.

### 2.5. Conceptual Framework Development

The conceptual framework was developed iteratively from the thematic synthesis by integrating evidence on structural drivers, food-system governance, dietary exposures, and oral-health pathways. The proposed relationships were refined through comparison with existing food-security, sustainability, and One Health perspectives, with particular attention to conceptual gaps and the underrepresentation of oral health. The resulting framework represents an evidence-informed conceptual synthesis rather than a validated causal model. It aims to organize current knowledge, clarify potential pathways linking food-system structures with oral-health outcomes, and identify priorities for future interdisciplinary research.

## 3. Financialization and the Structural Transformation of Contemporary Food Systems

Financialization has progressively transformed food and agricultural commodities from goods produced and exchanged primarily for consumption into assets increasingly embedded within global financial markets [15,16]. Although commodity markets perform established functions in price discovery and risk management, the increasing influence of financial institutions, investment strategies, and actors primarily seeking returns from price movements has altered the organization and governance of contemporary food systems [15,16,19]. The historical development of global commodity markets is closely associated with colonial trade and the geographical reorganization of agricultural production and supply chains [21,22]. In recent decades, however, financialization has increased the distance between investment decisions and the material conditions of food production, producer livelihoods, and food access [56]. Commodity speculation and the growing participation of financial actors with limited involvement in physical food production or delivery have further reinforced concerns regarding price volatility, market transparency, and the distribution of risks and economic value across food systems [19,63]. These processes extend beyond commodity trading and contribute to broader transformations in food-system organization, including corporate concentration, consolidation of market power, and increasing influence of financial interests over agricultural production and investment decisions [15,23]. The concentration of economic power among a limited number of corporations and financial actors may influence which commodities are produced and traded, how resources and risks are distributed across food supply chains, and whose interests are represented in food-system governance [56,57]. From a public-health perspective, these upstream structural processes remain comparatively underexamined. Research has more frequently focused on downstream food environments, retailers, and consumer behavior, while the influence of financial actors, commodity markets, and corporate concentration on dietary exposures and population health has received less attention [56,58]. Examining financialization as a structural determinant of food-system organization therefore provides an important basis for understanding how upstream economic processes may influence food environments, dietary patterns, health inequalities, and downstream health outcomes [15,16,58].

### 3.1. Modern Developments in Increased Financialization

The 2008 global financial crisis marked an important acceleration in food-system financialization. Rising food and oil prices, increased demand for agricultural commodities for biofuel production, and the growing participation of financial investors intensified speculative activity in commodity markets [63]. Between 2008 and 2012, food prices increased substantially in the United Kingdom, while global maize prices rose sharply, illustrating how volatility in international commodity markets can be transmitted to domestic food prices without generating proportional benefits for agricultural producers [64,65]. The growing participation of non-commercial financial actors further strengthened the treatment of food commodities as investment assets, increasing the influence of financial markets over price formation and the distribution of value across food supply chains [66,67]. These developments also exposed a fundamental tension between global commodity markets and food security. Financial speculation increased markedly during this period, while the assumption that market mechanisms would efficiently allocate food remained influential in national and international policy [68,69,70]. However, commodity flows respond primarily to purchasing power and expected returns rather than nutritional needs, meaning that increased global trade does not necessarily improve food access or producer livelihoods. Financialization may therefore amplify the separation between the economic value of food commodities and their social [68,69,70] function as essential resources for human nutrition and food security [67,68]. Subsequent crises have demonstrated how geopolitical instability and disruptions to global supply chains can intensify these vulnerabilities. Governments increasingly use food trade, agricultural investment, and infrastructure development as instruments of geopolitical influence, as illustrated by Chinese foreign investment strategies and Russian grain diplomacy [71,72]. More recently, the COVID-19 pandemic and the war in Ukraine exposed the fragility of globally integrated food markets through export restrictions, supply-chain disruptions, labor shortages, and sharp increases in grain prices. These events demonstrate that financialized and highly interconnected food systems may transmit economic and geopolitical shocks rapidly across countries, with disproportionate consequences for import-dependent and economically vulnerable populations.

### 3.2. Financialization, Market Concentration, and Producer Power

The expansion of financialization has altered the distribution of economic value and decision-making power within global food systems. Although commodity futures and hedging mechanisms can support price discovery and risk management, the growing participation of non-commercial investors, algorithmic trading, and commodity index funds has increasingly connected agricultural commodities with broader financial markets [15]. Clapp reported substantial growth in speculative participation between 1996 and 2011, accompanied by the entry of financial actors with limited involvement in the physical production, distribution, or delivery of food [15]. Financialization therefore extends beyond commodity trading to encompass processes through which profits are increasingly generated from financial transactions, asset appreciation, and market positioning rather than from agricultural production itself [15,56]. These processes operate within highly concentrated commodity markets. A limited number of global trading corporations control substantial shares of international grain and agricultural commodity flows, giving them considerable influence over supply chains, market information, pricing, and value distribution [57]. Market concentration may generate efficiencies and facilitate large-scale coordination, while futures markets can provide mechanisms for managing production risks [19,57]. However, unequal access to financial instruments, infrastructure, storage capacity, market information, and bargaining power means that the benefits and risks of commodity markets are unevenly distributed across food-system actors [15]. These asymmetries are particularly important for small-scale producers. Farms below two hectares produce approximately one-third of the global food supply and remain central to local food systems, rural livelihoods, and food security [73,74,75]. Smallholders also contribute substantial proportions of food production in regions where agriculture remains a major source of employment and income, particularly in low- and middle-income countries [75,76,77,78]. Nevertheless, their importance to food production does not necessarily translate into proportional control over markets, productive resources, or the economic value generated throughout global supply chains [57,73,74,75,76,77,78].

Producer vulnerability is strongly influenced by access to infrastructure and market resources. Unlike larger agricultural enterprises with storage facilities, financial reserves, market information, and greater capacity to delay sales, many smallholders must sell commodities shortly after harvest and are therefore more exposed to price volatility and unfavorable bargaining conditions [76,77,78,79]. These constraints are evident among coffee and cocoa producers and are not confined to low-income countries, as dairy and livestock farmers in higher-income economies may also face declining producer margins and concentrated purchasing power among processors and retailers [76,77]. Farm size alone, however, is an insufficient measure of producer vulnerability because its economic significance varies across agricultural systems, commodities, geographical contexts, and institutional arrangements [75,79]. Small- and medium-sized farms may depend on unpaid family labor, off-farm employment, remittances, cooperatives, and other income-diversification strategies to remain economically viable [71,72,73,74,75,76,77,78,79]. Conversely, agricultural modernization and increasing farm sizes do not necessarily guarantee profitability when producers remain dependent on concentrated input suppliers, processors, traders, and retailers [57,79].

Financialization and market concentration can be understood as interconnected processes that influence who controls productive resources, who can manage market risks, who captures value, and who bears economic vulnerability within food systems [15,56,57]. The central issue is not simply whether agricultural production becomes more efficient or globally integrated, but whether producers retain sufficient bargaining power, economic resilience, and agency within increasingly concentrated and financially oriented food systems [57,73,74,75,76,77,78]. These asymmetries provide an important basis for examining food-system inequity and the subsequent role of food sovereignty as a governance response.

### 3.3. Financialization, Land Concentration, and Agribusiness Consolidation

Financialization extends beyond commodity markets to the ownership of agricultural land and agribusiness assets. Farmland has increasingly attracted institutional investors, corporations, and wealthy individuals because it can generate returns through land appreciation, agricultural production, subsidies, and portfolio diversification. At the same time, technological intensification and rising capital requirements have contributed to farm consolidation, as larger enterprises are better positioned to invest in machinery, digital technologies, and infrastructure than smaller producers [80]. These processes may increase productivity but can also widen economic inequalities between farms and reinforce the concentration of productive resources. The consolidation of agricultural production is closely connected with mergers, acquisitions, and increasing corporate control across agri-food value chains. Smaller farms that cannot sustain the capital investments required for technological modernization may be absorbed by larger enterprises or exit agricultural production altogether, contributing to declining farmer numbers and increasing disparities in agricultural income [80]. Financialization therefore influences not only the organization of agricultural production but also access to land, technology, subsidies, and the capacity to remain economically viable within increasingly capital-intensive food systems.

Transnational land acquisitions represent another manifestation of these processes. Governments, corporations, and investment funds have acquired or leased large areas of agricultural land outside their countries of origin to secure food supplies, diversify investments, and expand control over agricultural production [81]. Such acquisitions may generate infrastructure, employment, and investment in agricultural development; however, they may also displace smallholders, weaken customary land rights, consolidate fragmented farms, and redirect agricultural production toward export markets rather than local food needs [82]. These dynamics are particularly relevant for countries with limited agricultural resources or high dependence on food imports. Strategies combining overseas agricultural investment, acquisition of food companies, technological innovation, and transnational supply-chain development have been used to strengthen national food availability and reduce exposure to domestic production constraints [83,84]. However, the geographical location of investment capital does not necessarily correspond to the nationality of its ultimate beneficiaries, reflecting the increasingly complex and transnational organization of agricultural finance and ownership. The consequences of land financialization extend beyond changes in property ownership. Rising farmland prices, farm consolidation, technological substitution of agricultural labor, and concentration of control over land and natural resources may create barriers to entry for new farmers and reduce the economic viability of smaller producers [80,81,82]. In some settings, local producers may shift from independent agricultural production to waged labor or migrant workers, while export-oriented production can weaken the relationship between local productive resources and local food security [81,82]. Financialization of land and agribusiness can result in a process that redistributes ownership, control, economic value, and decision-making power within food systems. Its significance lies not simply in increasing agricultural investment but in determining who can access productive resources, who benefits from technological and financial transformation, and whether agricultural development strengthens or weakens producer agency and local food security [80,81,82,83,84]. These processes reinforce the broader concentration of power within contemporary food systems and provide an important structural link between financialization, inequality, and the subsequent discussion of food sovereignty.

### 3.4. Financialization and Inequity

The financialization of food raises important questions concerning the distribution of economic value, risk, and decision-making power across food systems. A central distinction lies between food as a commodity traded for financial return and food as a social and ecological commons essential to human well-being [1,15,26]. Although futures markets may support price discovery and enable producers to manage risk, their structure and scale primarily favor actors capable of accessing large-volume commodity markets and sophisticated financial instruments [19]. Small-scale producers often participate from weaker bargaining positions and capture only a limited share of the final value generated within global commodity chains, despite their substantial contribution to the production of commodities such as coffee and cocoa. These asymmetries have become more pronounced as commodity markets have attracted institutional investors, hedge funds, banks, and other financial actors with limited involvement in physical food production [15,26]. The resulting concentration of financial and market power may increase the distance between investment decisions and their consequences for producer livelihoods, food access, and population health.

Financialization therefore has important implications for equity because the economic returns and risks generated within food systems are unevenly distributed among investors, corporations, producers, and consumers [15,26,38]. Finally, these findings also reveal the limits of evaluating food security primarily through availability, access, utilization, and stability. The inclusion of agency and sustainability recognizes that food-system performance also depends on who controls productive resources, participates in decision-making, captures economic value, and bears the social and environmental consequences of food-system organization [52]. Financialization and food sovereignty are therefore analytically interconnected: the former explains processes through which economic power and control may become concentrated, whereas the latter examines whether producers and communities retain meaningful agency over food production, distribution, and governance [52,69,73]. Importantly, these relationships are conceptual and may operate differently across food-system contexts, governance arrangements, and socioeconomic settings. They also provide the conceptual transition to the following section on food sovereignty as an alternative governance perspective within contemporary food systems.

## 4. Global Trade and Sovereignty: Food Security at the National and Household Levels

Food sovereignty provides a rights-based governance framework that shifts attention from aggregate food availability toward the distribution of power, resources, and decision-making capacity within food systems [23,85]. It asserts that producers, communities, and consumers should have meaningful influence over food production, distribution, and consumption and the capacity to define food and agricultural priorities according to their social, cultural, ecological, and economic contexts [85]. Core principles include the protection of local and domestic food production, control over productive resources, the capacity to determine appropriate levels of self-reliance, resistance to unfair trade practices and market dumping, and trade arrangements that support equitable producer livelihoods [85]. This perspective is consistent with evidence on the social determinants of health, demonstrating that food insecurity, malnutrition, and health inequalities arise from structural conditions rather than individual behaviors alone [24]. Trade agreements and agricultural policies can influence food availability, dietary environments, and health by altering domestic production, market access, food prices, and the regulatory capacity of governments [25]. Agricultural and economic policies may also reproduce inequalities when policy benefits and productive resources are disproportionately captured by larger and more powerful agricultural actors [26]. Indigenous and local food systems further demonstrate how disruptions to land access, traditional food practices, and community control over productive resources can contribute to nutritional and health inequalities [27,31,33].

Food-system transformation therefore requires attention to the structural conditions that shape dietary opportunities and population health [28,32]. Food insecurity and nutritional vulnerability are produced through interactions among income inequality, political marginalization, environmental conditions, market structures, and unequal access to health-supportive resources [24,28,29,30,31,32,33]. These determinants operate across local, national, and global scales and may accumulate among populations already experiencing socioeconomic disadvantage [29,30]. Commercial actors further influence food environments through product formulation, pricing, marketing, lobbying, and other political practices that shape both dietary exposures and regulatory responses [58].

Food sovereignty extends this structural perspective by integrating the right to adequate and nutritious food with the right to participate in determining how food systems are organized and governed [23,85]. International social movements and civil-society organizations have contributed to the development of this rights-based approach by challenging food-system models characterized by corporate concentration, industrialized production, and unequal control over land and natural resources [85]. Their contribution is particularly important because increased agricultural productivity or technological efficiency does not necessarily improve food security when economic value, productive assets, and decision-making power remain concentrated among dominant market actors [26,58,85]. Access to land, water, fisheries, biodiversity, and other productive resources is consequently central to both food sovereignty and equitable food-system governance [56,60,62,64]. Public support for producers, fairer trade arrangements, protection from market dumping, and stronger bargaining capacity may improve the ability of small- and medium-scale producers to maintain viable livelihoods and participate meaningfully in food-system decisions [25,26,85]. Gendered inequalities in land ownership, agricultural labor, access to credit, and political representation further demonstrate that food sovereignty depends on the distribution of both material resources and decision-making power within households, communities, and institutions [29,30,85]. Food sovereignty also raises questions concerning the governance and effectiveness of international food assistance. External interventions may have limited long-term effects when they address immediate food deficits without strengthening local productive capacity, livelihoods, institutional participation, and community agency [31,33,85]. The increasing involvement of private foundations and corporate philanthropy in food-system governance introduces additional questions concerning transparency, accountability, priority setting, and the transfer of influence from public institutions toward private actors [58,85]. These developments reinforce the need to evaluate food-system interventions not only according to the resources they mobilize but also according to who controls decision-making, whose interests shape policy priorities, and whether affected populations gain meaningful agency [23,58,85,86]. Food sovereignty should therefore not be interpreted as complete national self-sufficiency or the rejection of international trade [23,85]. Food sovereignty represents a multilevel governance approach concerned with control over productive resources, participation in decision-making, cultural and ecological appropriateness, equitable livelihoods, and the distribution of food-system benefits and risks [23,25]. This distinction is central to the present review: whereas financialization explains how ownership, market power, and value extraction may become concentrated, food sovereignty provides an analytical framework for examining whether producers and communities retain sufficient agency to influence the food systems shaping their livelihoods, dietary environments, and health outcomes [23,85].

### 4.1. Food Sovereignty, Trade Liberalization, and the Redistribution of Food-System Power

Government food-aid policies and international development interventions have increasingly been examined through the lens of food sovereignty. In Haiti, Steckley et al. (2023) [86] discussed a food-sovereignty policy that differed substantially from previous approaches by prioritizing smallholder farming, encouraging the production and consumption of traditional foods, and protecting domestic agricultural production from import competition. More than USD 150 million was subsequently allocated to the Emergency Resilient Agriculture for Food Security Project (PARSA) [87], which was presented as closely aligned with the plans of the Ministry of Agriculture, Natural Resources and Rural Development (MARNDR) and the broader policies and strategies of the Government of Haiti [88].

The relationship between trade liberalization, food security, and food sovereignty remains contested. Trade liberalization may increase food availability and access through cheaper imports, but it can also undermine domestic agricultural production, producer livelihoods, and national capacity to shape food systems [86]. International organizations have generally emphasized the potential contribution of economic liberalization and trade to growth and food availability, whereas critics argue that such approaches may insufficiently address the structural causes of food insecurity and unequal distributions of food-system power. The Food and Agriculture Organization (FAO) similarly recognizes that relationships among trade liberalization, market imbalances, land access, and food security are complex and that reforms implemented in one country may generate unintended consequences elsewhere [89]. The International Monetary Fund, World Bank, and FAO have frequently emphasized poverty, conflict, disease, climate change, food waste, and insufficient agricultural investment as determinants of food insecurity, while giving less attention to the political-economic processes that produce and reproduce these conditions [59]. However, all this takes place at a time when major institutions are rowing back on commitments to poverty reductions, climate change, etc. Addressing food insecurity and malnutrition therefore requires state policies capable of integrating economic, environmental, social, cultural, trade, and health dimensions rather than treating poor diets as isolated outcomes of individual behavior or insufficient food production [90,91]. Food sovereignty provides such a multidimensional perspective by explicitly addressing the distribution of decision-making power within food systems.

The concept of food sovereignty defined by the global peasant movement La Via Campesina in 1996 [92] as the determination of agricultural and food policies as a right of peoples and recognizes their right to healthy and culturally appropriate food produced through ecologically sustainable methods [85]. Food sovereignty therefore seeks to decentralize food-system power and establish more diverse, democratic, and sustainable systems of food production and provision while reducing dependence on globalized corporate food regimes [60]. Placing small producers and consumers at the center of food systems requires explicit consideration of the effects of dominant food regimes on food insecurity, equity, and producer livelihoods [59,60]. Food-sovereignty approaches emphasize closer relationships between sustainable food production and producers, greater reliance on agroecological principles, and the development of more localized and circular food systems [93]. Such approaches may provide pathways toward improved community health and greater health equity by connecting food-system governance with social and environmental determinants of health [59,61]. However, the implementation of food sovereignty is constrained by the concentration of political and economic power within national and global food systems [56]. Dominant industrial production systems may reinforce dietary inequalities through intensive input dependence, limited agricultural diversity, unequal control over productive resources and lobbying [90,91]. Redistributing agency toward producers and communities consequently challenges the political and economic structures of the global agro-industrial system [94]. Food sovereignty should therefore be understood as an evolving and contested governance process whose implementation varies according to institutional and political contexts [95,96].

Scaling food-sovereignty initiatives introduces additional governance challenges because their development may be facilitated or constrained by state institutions, regulatory systems, and access to public resources [96,97]. Food-sovereignty movements may need to cooperate with governments that simultaneously provide funding and policy opportunities while maintaining institutional arrangements that reproduce existing inequalities [97]. Food sovereignty has been incorporated into constitutional or policy frameworks in Bolivia, Ecuador, Venezuela, Senegal, Mali, and Nepal, providing important contexts for examining the contradictory role of the state, the influence of peasant mobilization, and the institutionalization of food-sovereignty principles [95,97,98,99]. These debates also extend to land rights, gender equality, and cultural autonomy.

Food sovereignty encompasses the rights of peasant families, control over agricultural land, respect for women’s rights, cultural practices, and locally determined food systems [100]. In many settings, those who cultivate agricultural land do not own it and instead depend on tenancy arrangements, crop-sharing agreements, or other forms of unequal access to productive resources, further complicating the effective redistribution of food-system power [101].

### 4.2. Food Sovereignty Under Climate, Development, and Sustainability Pressures

Climate change represents an additional constraint on food sovereignty, food security, and sustainability because it affects food availability, accessibility, utilization, and affordability [101]. Rising temperatures, changing precipitation patterns, and extreme climatic events influence crop yields and increase the vulnerability of agricultural production systems [102,103,104]. Strengthening food sovereignty therefore requires policies that combine agricultural productivity with climate-resilient farming practices, efficient resource use, and reduced dependence on vulnerable production systems [105]. The implications of climate vulnerability are context-specific. In Egypt, persistent wheat-import dependence, water scarcity, degraded agricultural land, increasing global food prices, and projected reductions in cereal yields demonstrate the importance of crop diversification and improved irrigation efficiency [105]. More broadly, agricultural production systems range from technologically intensive and climate-controlled models to semi-intensive, small-scale, and agroecological systems that remain important for rural livelihoods and local food sovereignty [106,107,108].

Agricultural modernization may improve productivity, biosecurity, animal welfare, and production efficiency, but it generally requires substantial investment in infrastructure and technology and may increase capital dependence among producers [109,110,111,112]. Alternative production systems, including free-range and agroecological farming, may reduce environmental pressures, support animal welfare, and align more closely with sustainability objectives, although their performance depends on local ecological, economic, and institutional conditions [107,112,113]. The competitiveness and sustainability of agricultural production should therefore be evaluated in relation to the capacity of production systems to adapt to local environmental and socioeconomic conditions [112,114]. China illustrates the tensions between competing pathways of food-system transformation. One perspective supports state-led agricultural modernization and large-scale agribusiness as mechanisms for increasing production and strengthening food security, whereas critics argue that these models may intensify food-safety concerns, socioeconomic inequalities, and the marginalization of small-scale producers [115,116,117,118,119]. An alternative perspective emphasizes stronger support for smallholder agriculture and the social organization of food production, distribution, and consumption to limit excessive dependence on capital-intensive agricultural development [20,21,22,23,24,25,26,27,28,29,30,31,32,33,34,35,36,37,38,39,40,41,42,43,44,45,46,47,48,49,50,51,52,53,54,55,56,57,58,59,60,61,62,63,64,65,66,67,68,69,70,71,72,73,74,75,76,77,78,79,80,81,82,83,84,85,86,87,88,89,90,91,92,93,94,95,96,97,98,99,100,101,102,103,104,105,106,107,108,109,110,111,112,113,114,115,116,117,118,119,120,121,122]. Nevertheless, the development of a corporate-centered food-security governance system and restrictions on civil-society participation raise questions regarding the extent to which contemporary agricultural transformation supports meaningful food sovereignty [123]. More broadly, the implementation of food sovereignty depends on whether governance reforms genuinely redistribute power, transform social relationships, and facilitate meaningful participation within existing food systems [124]. Structural transitions may also be constrained by established institutions and dominant systems of knowledge production and dissemination, as demonstrated by the development of organic agriculture in the United Kingdom [125].

Food sovereignty may be understood as a multilevel and context-dependent process in which local initiatives interact with institutional structures and broader political-economic conditions. Its implementation is closely connected with the Sustainable Development Goals (SDGs), particularly SDG2, which links the eradication of hunger and improved nutrition with the promotion of sustainable agriculture [126]. Achieving these objectives requires managing trade-offs among increasing food production, reducing agriculture-related environmental pressures, and adapting food systems to climate change [125,126,127,128,129,130]. In this sense, agri-food transformation must account for synergies and tensions between SDG2 and broader sustainability objectives rather than addressing food production, food security, and environmental protection as independent policy domains [131]. Cross-national evidence further demonstrates that food sovereignty is associated with social, demographic, and environmental conditions extending beyond conventional measures of food security [132]. Effective food-system transformation consequently requires coordinated action across governance levels and sectors, with explicit consideration of interactions, synergies, and trade-offs among food security, social equity, and environmental sustainability [62].

Although food sovereignty and financialization are commonly discussed in relation to agricultural production, trade, and food security, their consequences ultimately materialize through population diets and health outcomes. The availability, affordability, promotion, and consumption of foods within particular food environments are shaped by these upstream structural and governance processes. Accordingly, oral health may provide a measurable biological pathway through which the consequences of financialization, food sovereignty, and food-system transformation become visible, linking upstream food-system governance with downstream health outcomes.

## 5. Health, Oral Health, Food-System Performance and One Health

The relationship between food-system transformation, food sovereignty, financialization, and health remains insufficiently conceptualized within contemporary food-security research. While considerable attention has been devoted to agricultural productivity, sustainability, food access, and nutritional adequacy, emphasis has also been placed on understanding how food-system structures ultimately translate into measurable biological outcomes. Recent developments in food-security theory have expanded the conceptual framework beyond the traditional dimensions of availability, access, utilization, and stability to include agency and sustainability, thereby recognizing that food systems should be evaluated not only according to their capacity to provide food, but also according to their ability to promote equitable, resilient, and health-supportive environments [54,55].

Within this broader framework, oral health offers a particularly useful analytical lens because it occupies a unique position at the interface between food systems and human biology. Food-system decisions influence agricultural production, food processing, market structures, retail environments, and dietary behaviors, all of which ultimately affect oral microbial ecology, oral disease development, and population health outcomes [44]. Oral health therefore provides a measurable biological endpoint through which the consequences of food-system transformation can be assessed [45]. This perspective allows oral diseases to be interpreted not solely as clinical conditions or behavioral outcomes, but also as manifestations of broader social, economic, environmental, and political processes operating throughout food systems [46,47].

The following sections examine this relationship through three complementary perspectives. First, food-system financialization and the expansion of ultra-processed foods are explored as major drivers of dietary transformation. Second, oral health is considered an indicator of food-system performance. Finally, oral health is situated within a broader One Health framework that integrates human, environmental, and food-system sustainability. Together, these perspectives position oral health as a biological bridge connecting food-system governance, dietary transformation, sustainability, and health equity.

### 5.1. Financialization, Ultra-Processed Foods and the Nutrition Transition

The public-health relevance of financialization lies in its capacity to influence which foods become economically attractive to produce, market, and distribute at scale. Financialized business models prioritize market expansion, asset growth, and shareholder returns, creating incentives for products capable of generating high margins, repeated consumption, brand differentiation, and global scalability [34,35]. Ultra-processed foods (UPFs) are particularly compatible with this economic logic because inexpensive commodity-derived ingredients can be transformed through processing, formulation, branding, and marketing into standardized products with long shelf lives and substantial added value [34,37,38,39]. The global expansion of UPFs should therefore be interpreted not only as a consequence of urbanization or changing consumer preferences but also as an outcome of structural changes in food production, trade, retail, and corporate organization [36,37,40]. Trade liberalization, supermarketization, vertically integrated supply chains, market concentration, and intensive marketing have increased the availability, affordability, visibility, and desirability of highly processed products across diverse socioeconomic contexts [36,37,41,42,43]. Dietary behavior consequently occurs within commercially structured environments in which the range and relative attractiveness of available foods are shaped by corporate strategies and unequal market power rather than by autonomous consumer preferences alone [37,43,58].

This interpretation extends the analysis of UPFs beyond their nutritional composition. Wood et al. (2023) [34] conceptualized UPFs as vehicles of financial value extraction through which corporations convert relatively inexpensive agricultural inputs into differentiated and highly profitable products using processing, branding, intellectual property, portfolio diversification, and global market expansion. The significance of this process is that financialization may affect population health indirectly by reorganizing corporate incentives and food environments, thereby influencing the distribution, intensity, and persistence of dietary exposures [34,37,43]. The relationship between financialization and health is therefore neither direct nor deterministic; it operates through intermediate commercial and structural mechanisms that shape the conditions under which dietary patterns are produced.

These mechanisms also have implications for agency and sustainability. Contemporary food-security frameworks recognize that food-system performance depends not only on availability, access, utilization, and stability but also on the capacity of populations to participate in food-system decisions and on the preservation of ecological and social foundations over time [23,54]. Food environments dominated by concentrated corporate interests, intensive marketing, and widespread availability of UPFs may constrain meaningful dietary agency by structuring the range, price, accessibility, and promotion of available foods [36,43,54]. At the same time, UPF production frequently relies on intensive commodity agriculture, extensive processing, global supply chains, transportation, and packaging, raising broader concerns regarding resource use, environmental pressures, and the compatibility of financialized food systems with long-term sustainability objectives [37,38,55].

The health implications of these processes emerge through the cumulative dietary exposures generated within such food environments. UPFs frequently contribute to dietary patterns characterized by high exposure to free sugars, refined carbohydrates, sodium, industrial fats, acidic products, and frequent eating occasions [37,48,49,50,51]. Financialization may therefore become biologically relevant through a multilevel pathway in which financial and corporate incentives influence market concentration and product portfolios; these processes reshape food environments and dietary exposures; and repeated exposures interact with behavioral, socioeconomic, microbial, and host-related factors to influence disease risk [34,37,43,58]. This pathway provides a more analytically defensible interpretation than attributing health outcomes directly to financialization because it identifies the intermediate mechanisms through which upstream structural processes may become biologically embodied.

Within this pathway, oral health represents a particularly relevant biological interface because the oral cavity is repeatedly and directly exposed to food-system outputs. Diets characterized by frequent consumption of free sugars, refined carbohydrates, acidic products, and UPFs may alter oral microbial ecology, promote cariogenic conditions and demineralization, contribute to erosive processes, and interact with inflammatory pathways relevant to periodontal health [44,45,48,49,50,51]. Oral-health outcomes may therefore provide a measurable biological interface through which structurally patterned food environments and cumulative dietary exposures can be investigated. However, because oral diseases are multifactorial and influenced by preventive behaviors, fluoride exposure, socioeconomic conditions, healthcare access, and biological susceptibility, they should be considered potential complementary indicators rather than specific measures of food-system performance [44,45,46,47].

**Figure 1:**
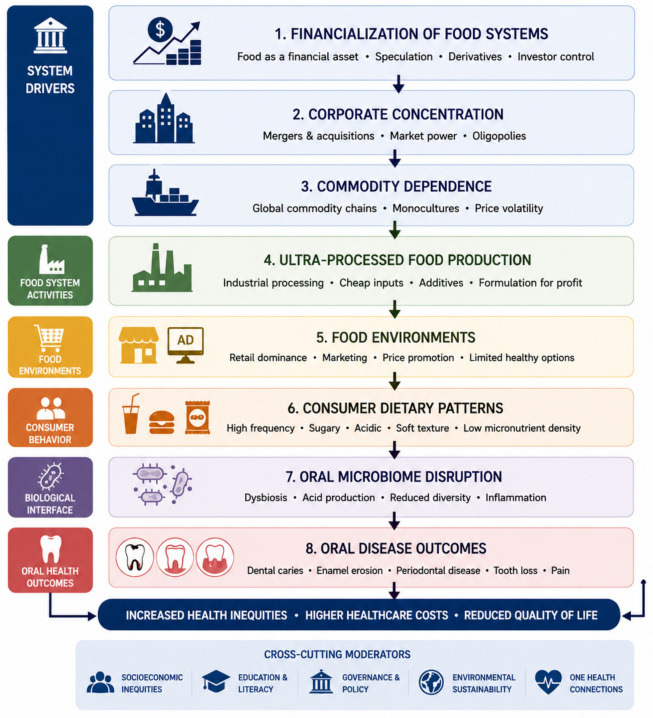
Conceptual framework linking food-system financialization with oral-health outcomes. Financialization may influence corporate concentration, commodity dependence, UPF expansion, food environments, dietary exposures, and oral-health outcomes through interconnected structural and biological pathways. The framework positions oral health as a potential biological interface within food-system sustainability and One Health research. (Created in BioRender. Antoniadou, Μ. (2026) https://BioRender.com/1o4tr07 accessed on 29 July 2026).

The resulting pathway—financialization and corporate power → food-system organization → UPF expansion and commercially structured food environments → population dietary exposures → biological mechanisms → oral-health outcomes—provides the analytical basis for the proposed conceptual framework presented in Figure 1. Rather than implying a direct causal relationship between financialization and oral disease, the framework identifies intermediate mechanisms, modifying conditions, and empirically testable relationships through which upstream food-system structures may contribute to downstream health inequalities.

### 5.2. Oral Health as a Potential Indicator of Food-System Performance

Conventional assessments of food-system performance emphasize availability, affordability, productivity, and food security but provide limited information on the cumulative biological consequences of prevailing food environments [54,133,134]. Oral health may complement these measures because the oral cavity is repeatedly exposed to dietary inputs and represents a biological interface through which food composition, processing, and consumption patterns interact with microbial ecosystems and host responses [46,47,135]. This perspective is particularly relevant to food environments characterized by widespread availability and consumption of ultra-processed foods (UPFs). Dietary patterns high in free sugars, refined carbohydrates, acidic products, and highly processed foods may promote ecological shifts in the oral microbiome, enamel demineralization, erosive processes, and inflammatory pathways associated with caries and periodontal disease [37,48,49,50,51]. Population-based studies have associated UPF consumption with early childhood caries and periodontitis, while systematic evidence supports an association with dental caries among children and adolescents [49,50,51]. More recent cohort evidence further suggests that these relationships may involve both direct and indirect pathways [136].

The significance of these findings extends beyond individual dietary behavior. Oral diseases are socially patterned and arise from interactions among cumulative dietary exposures, socioeconomic conditions, preventive resources, healthcare access, and biological susceptibility [46,137]. Their distribution may therefore provide information on how food environments and structural inequalities become biologically embodied across populations. However, because oral diseases are multifactorial, they should not be interpreted as specific measures of food-system performance but as potential complementary indicators alongside nutritional, environmental, socioeconomic, and health measures. Measures such as caries experience, periodontal disease burden, erosive tooth wear, tooth loss, and oral-health-related quality of life could contribute to evaluating whether food systems support health and equity in addition to food availability and economic productivity [44,48,54]. From a food-sovereignty perspective, population oral-health patterns may also help investigate whether differences in agency, access to nutritious foods, and control over food environments are associated with measurable health inequalities. Incorporating oral-health outcomes into food-system assessment frameworks therefore represents an empirically testable proposition that requires longitudinal, multilevel, and comparative validation before oral health can be established as a formal indicator of food-system performance.

### 5.3. Oral Health Within a One Health Framework

One Health provides a systems-based framework for understanding health outcomes as products of interactions among human, animal, environmental, and ecosystem health [52,138]. Its contemporary scope extends beyond infectious diseases to encompass the biological, ecological, social, economic, and political processes that shape population health [52,139]. Food systems constitute a critical domain of these interactions because agricultural production, environmental change, food processing, dietary exposures, microbial ecosystems, and human health are dynamically interconnected [54,55]. Despite these interdependencies, oral health remains insufficiently represented in One Health research and policy [47,53]. This omission is important because oral diseases share structural determinants with other noncommunicable diseases, including unhealthy dietary exposures, socioeconomic inequalities, commercial influences, environmental conditions, and unequal access to preventive resources and healthcare [46,47,135]. Moreover, the oral ecosystem is continuously exposed to dietary inputs, and changes in food environments may influence microbial ecology, host responses, and disease risk. Oral health therefore provides a relevant biological domain for investigating how food-system and environmental transformations become embodied within human populations [47,135]. Within this perspective, the significance of food-system financialization lies in the intermediate pathways through which economic structures may affect health. Financial and corporate incentives can influence agricultural production, product portfolios, food processing, marketing, and food environments, thereby shaping population dietary exposures [34]. Dietary patterns increasingly characterized by ultra-processed foods may subsequently contribute to oral microbial dysbiosis, cariogenic exposures, inflammatory processes, and adverse oral-health outcomes [50,51,134]. One Health thus provides a framework for connecting upstream economic and environmental processes with intermediate food-system changes and downstream biological consequences without implying a direct or deterministic relationship between financialization and oral disease.

The environmental dimension further strengthens this integration. Climate change, biodiversity loss, agricultural intensification, and resource depletion may affect food-system resilience, dietary quality, and population health, while sustainable dietary transitions can generate both environmental and health-related benefits and trade-offs [52,140]. Oral-health outcomes may contribute to evaluating these transitions because dietary changes can alter cariogenic and erosive exposures, nutritional adequacy, microbial ecology, and oral-disease risk [47,137]. However, their contribution should be assessed alongside established environmental, nutritional, and population-health indicators rather than treated as a specific measure of sustainability. One Health also introduces an ethical and governance dimension based on interdependence, shared vulnerability, and collective responsibility [139]. This perspective intersects with food sovereignty, which emphasizes agency, equitable participation, ecological sustainability, and greater community influence over food-system governance [23,54,59]. Integrating these perspectives suggests that the health consequences of food systems depend not only on environmental conditions and dietary exposures but also on the distribution of power and decision-making capacity. Populations differ in their ability to influence food environments, access nutritious and culturally appropriate foods, and avoid commercially structured dietary exposures, potentially contributing to persistent oral-health inequalities [36,46,54]. So, the proposed integration extends One Health analysis by positioning oral health as a potential biological interface within a multilevel food-system network rather than as an isolated clinical endpoint or validated indicator of food-system performance. Structural and governance conditions influence food-system activities and environments; these shape dietary exposures; dietary exposures interact with microbial, behavioral, socioeconomic, and host-related factors; and the resulting oral-health outcomes may provide information on the biological embodiment of food-system inequalities [46,47,135]. It is important to mention that these relationships may operate through feedback mechanisms: population health burdens can influence healthcare costs, policy priorities, public demand, and regulatory responses, which may subsequently affect food-system governance and commercial practices.

**Figure 2:**
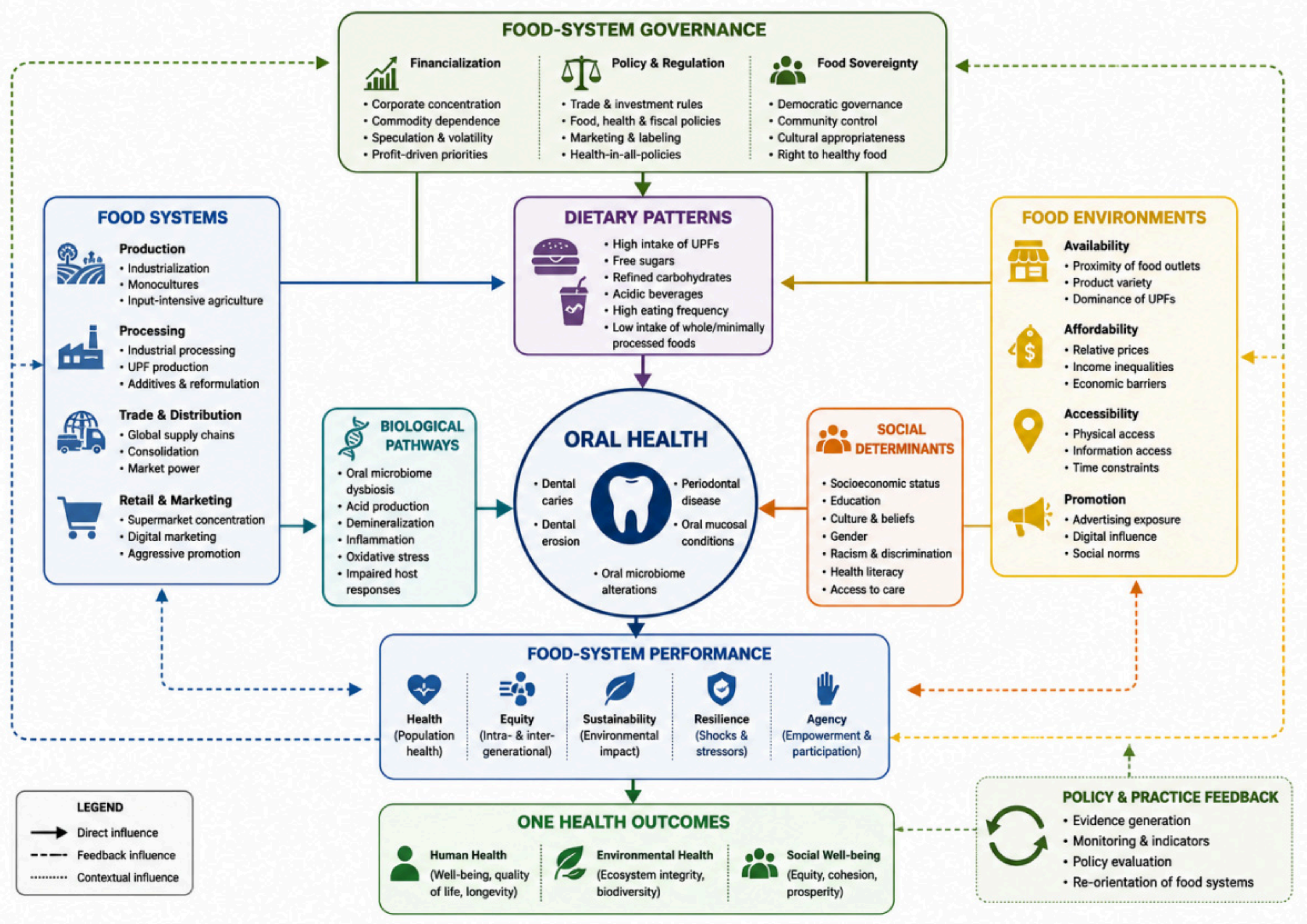
System model positioning oral health within the food-system–One Health nexus. The framework illustrates the interactions among food-system governance, food systems, food environments, dietary patterns, biological pathways, oral-health outcomes, and One Health outcomes. Oral health is conceptualized as both an outcome and a feedback indicator of food-system performance, linking upstream governance processes with downstream biological and societal consequences. (Created in BioRender. Antoniadou, Μ. (2026) https://BioRender.com/1o4tr07 accessed on 29 July 2026).

Accordingly, the proposed framework should be interpreted as a dynamic, hypothesis-generating systems model rather than a linear causal pathway. Its contribution lies in integrating food-system governance, financialization, agency, environmental sustainability, dietary exposures, biological responses, and oral health within a common One Health perspective and in identifying relationships that can be empirically tested across populations and food-system contexts [47,52,53]. This systems-oriented interpretation is illustrated in Figure 2.

## 6. Discussion

The present review advances the interpretation of contemporary food systems by integrating financialization, food sovereignty, dietary transformation, and oral health within a common analytical framework. Contemporary food-security scholarship increasingly recognizes that availability, access, utilization, and stability are insufficient to capture the structural conditions that determine whether food systems are equitable, sustainable, and health-promoting, leading to the inclusion of agency and sustainability as additional dimensions of food security [23,54]. However, the biological consequences through which food-system organization becomes embodied in populations remain incompletely represented in these frameworks, particularly with respect to oral health [44,45,46,47]. The principal contribution of the present synthesis is therefore to connect upstream distributions of economic power and decision-making capacity with intermediate changes in food production, processing, markets, and food environments and, ultimately, with dietary exposures and oral-health outcomes [15,23,34,44,56]. This perspective extends food-system analysis beyond the parallel consideration of economic, environmental, and nutritional outcomes by proposing a multilevel pathway through which structural transformations may generate measurable biological consequences [45,46,47,54,55].

A central finding of the review is that financialization should not be interpreted exclusively as the increasing participation of financial actors in agricultural commodity markets. Rather, it represents a broader reorganization of food-system priorities around asset appreciation, shareholder value, market expansion, corporate consolidation, and financial returns [15,16,57]. Financialization may influence the allocation of capital, ownership of productive resources, corporate mergers and acquisitions, agricultural investment, commodity trading, and the strategic behavior of transnational food corporations [15,16,34]. These mechanisms are analytically important because they shape the economic incentives governing which commodities and food products are produced, processed, marketed, and globally distributed [34,56,57]. Accordingly, the public-health relevance of financialization lies less in a simple direct association between financial-market activity and disease than in its capacity to restructure the commercial and material environments within which population dietary exposures are generated [34,43,58]. This interpretation provides a more plausible account of the relationship between financialization and the global expansion of ultra-processed foods. Ultra-processed foods (UPFs) and products are particularly compatible with financialized business models because standardized formulations, inexpensive commodity-derived ingredients, extensive product differentiation, long shelf life, branding, and global scalability can facilitate market expansion and sustained returns on investment [34,36,37,38]. The increasing dominance of UPFs should therefore not be understood solely as a response to changing consumer preferences or urbanization, but also as the outcome of corporate strategies, trade liberalization, supermarketization, market concentration, and commercial practices that influence availability, affordability, marketing intensity, and consumption patterns [36,43]. This interpretation shifts part of the explanatory burden for unhealthy diets from individual behavior toward the political and commercial organization of food systems, consistent with broader evidence on the commercial determinants of health [37,43]. Nevertheless, the pathways connecting financialization with population health are complex and should not be represented as linear or deterministic. Financialization operates through intermediate mechanisms, including changes in corporate governance, investment priorities, market concentration, agricultural production, food processing, pricing, marketing, and retail environments, before influencing dietary exposures [15,56]. The health effects of these exposures are subsequently modified by socioeconomic position, cultural practices, education, healthcare access, regulatory environments, and biological susceptibility [24,25,26,27,28,29,30,31,32,33]. Financialization should therefore be conceptualized as an upstream structural determinant that modifies the distribution and intensity of health-related exposures rather than as an independent causal agent of specific diseases [15,58]. This distinction is theoretically important because it identifies multiple intervention points along the pathway and avoids reducing complex biological outcomes to a single economic process.

The synthesis further indicates that financialization and food sovereignty illuminate different but interdependent dimensions of food-system power. Financialization explains processes through which ownership, capital, market control, and economic value may become concentrated among increasingly powerful corporate and financial actors [15,57]. Food sovereignty, by contrast, directs attention to the distribution of agency, rights, productive resources, and decision-making authority among producers, communities, consumers, corporations, and governments [23,59,60,61]. Considering these perspectives together reveals that food-system inequalities are produced not only through unequal access to food but also through asymmetries in the capacity to determine what is produced, how food systems are organized, which forms of knowledge and production are supported, and whose interests are represented in governance processes [60,61].

Within the proposed framework, agency may therefore represent an important modifying dimension between structural food-system organization and downstream health outcomes. Greater participation in food-system governance, protection of small-scale producers, equitable access to land and productive resources, support for local and regional food systems, and improved access to culturally appropriate and nutritionally adequate foods may alter the environments within which dietary choices are made [61,73,75]. However, food sovereignty should not be idealized as an inherently health-promoting alternative to globalized food systems. Local food systems may reproduce socioeconomic inequalities, provide inconsistent access to nutritionally adequate foods, or operate within broader political and market structures that constrain meaningful redistribution of power [60,61]. Moreover, direct empirical evidence connecting food-sovereignty interventions with oral-health outcomes remains scarce. The proposed pathway from agency and governance to oral health should therefore be regarded as theoretically grounded but insufficiently tested, representing an important priority for future interdisciplinary research [59,61].

The integration of oral health constitutes the principal conceptual extension proposed by this review. Existing food-system frameworks primarily assess outcomes through food security, nutritional adequacy, environmental sustainability, economic performance, resilience, and population health [23,54,55,62]. Yet the oral cavity is the first biological environment repeatedly exposed to food-system outputs and provides a direct interface among dietary composition, eating frequency, salivary function, microbial ecology, host responses, and socially patterned exposures [44,45,46,47,48,49]. Frequent consumption of free sugars and refined carbohydrates promotes acidogenic and aciduric ecological shifts associated with dental caries, while acidic foods and beverages contribute to erosive tooth wear and dietary patterns associated with highly processed foods may interact with inflammatory and metabolic pathways relevant to periodontal health [45,48,49,50,51]. These mechanisms provide biological plausibility for incorporating oral-health outcomes into the analysis of food-system consequences [44,45]. More importantly, the proposed framework positions oral health as a potential marker of the biological embodiment of food-system inequalities. Food environments are socially patterned, and populations do not experience equivalent access to affordable, nutritious, minimally processed, and culturally appropriate foods [46,54,58]. Commercial exposure to heavily marketed, inexpensive, and highly processed products is also unequally distributed across socioeconomic groups and communities [37,43]. Because oral diseases accumulate through repeated exposures over time and are strongly socially patterned, their distribution may capture aspects of dietary inequality and structural disadvantage that are not fully represented by aggregate measures of food availability or economic productivity [44,45,50,51]. Oral-health inequalities may thus provide an additional analytical lens through which the downstream consequences of food-system organization can be investigated. This proposition, however, requires careful causal qualification. Dental caries, periodontal diseases, erosive tooth wear, tooth loss, and oral-health-related quality of life are multifactorial outcomes influenced by fluoride exposure, oral-hygiene practices, tobacco use, socioeconomic circumstances, healthcare access, comorbidities, medication use, behavioral factors, and biological susceptibility [44,45,46,47]. Oral-health outcomes cannot therefore be interpreted as specific or exclusive measures of food-system performance. Rather, their potential value lies in complementing established nutritional, socioeconomic, and environmental indicators by capturing biological responses to repeated dietary exposures within broader social contexts [46,47]. The proposed framework consequently conceptualizes oral health as a candidate biological indicator whose validity, sensitivity, specificity, and incremental value require empirical evaluation.

The temporal characteristics of oral diseases may further strengthen their potential relevance to food-system research. Many conventional food-security indicators measure current availability, affordability, consumption, or nutritional status, whereas oral diseases frequently reflect cumulative exposures occurring across extended periods of the life course [48,54,55]. Caries experience, tooth loss, periodontal destruction, and oral-health-related quality of life may therefore provide information on the longer-term embodiment of dietary and socioeconomic conditions [48,49,50,51]. Conversely, this cumulative nature complicates causal attribution because contemporary oral-health status may reflect historical food environments, preventive policies, healthcare systems, and earlier-life exposures. Longitudinal and life-course research will therefore be necessary to determine whether changes in food-system organization produce detectable changes in oral-health outcomes and over what temporal intervals such effects become observable [46].

The framework also contributes to the One Health literature by identifying oral health as a potentially informative but underdeveloped component of food-system analysis. One Health emphasizes the interdependence of human, animal, and environmental health and increasingly recognizes food systems as critical interfaces linking agricultural production, biodiversity, environmental degradation, antimicrobial pressures, microbial ecosystems, nutrition, and human health [52,55]. Oral health is relevant to this perspective because the oral ecosystem is directly exposed to dietary inputs and environmental influences while interacting with microbial communities, inflammatory processes, systemic health, and quality of life [47,53]. Integrating oral health into One Health research could therefore strengthen the analysis of pathways connecting agricultural and environmental conditions, food processing, dietary exposures, microbial ecology, and human health outcomes [47,53]. Under this scope, the inclusion of oral health within One Health should generate analytical and empirical value rather than merely broaden the terminology of existing frameworks. Demonstrating such value will require multilevel studies capable of linking macro-level food-system characteristics with meso-level food environments, individual dietary exposures, oral microbial or inflammatory pathways, and population oral-health outcomes [52]. Research should also examine whether oral-health indicators improve the explanatory, predictive, or monitoring capacity of existing food-system and One Health frameworks beyond established nutritional and noncommunicable-disease measures [44,45,46,47,54,55,62]. Without such empirical validation, the proposed framework should remain a hypothesis-generating conceptual synthesis rather than a validated monitoring model.

Furthermore, the findings have implications for food-system and public-health policy. Interventions focused primarily on nutritional education and individual behavior are unlikely to reduce diet-related inequalities when food environments remain shaped by concentrated corporate power, commercial practices, economic constraints, and unequal access to nutritious foods [43,58]. Policies addressing food formulation, marketing, fiscal measures, public procurement, competition, corporate accountability, and food access may therefore influence dietary exposures and related health outcomes [34,43]. Complementary governance strategies that strengthen producer and community participation, protect access to productive resources, and improve accountability may contribute to a more equitable distribution of food-system benefits and risks [23,59,61]. These implications also support greater participation of oral-health professionals in interdisciplinary food-system research and policy. Dentistry has traditionally emphasized clinical prevention, treatment, and individual risk modification, despite the contribution of commercial and social determinants to oral-health inequalities [44,45,46]. Engagement with food policy, sustainability, commercial determinants of health, and One Health could strengthen the contribution of oral-health research to population health and inform policies concerning sugar consumption, ultra-processed foods, marketing regulation, public procurement, and health-sensitive food-system assessment [34,46,47,50,53,58].

Future research should empirically test the multilevel pathways proposed in the framework. Longitudinal and comparative studies are needed to determine whether financialization, corporate concentration, and governance arrangements predict changes in food environments, dietary exposures, and oral-health outcomes across institutional contexts [15,34,57,59]. Natural experiments evaluating taxation, marketing regulation, public procurement, agricultural policy, competition measures, and local food-system interventions could strengthen causal inference [37,43]. Food-sovereignty initiatives should also be evaluated using nutritional and oral-health outcomes, while the incremental value of oral-health measures within food-system sustainability and One Health monitoring frameworks requires validation [60,61].

The evidence supporting these pathways remains uneven. Associations between dietary exposures and oral diseases are comparatively well established, and substantial evidence links corporate practices and food-system organization with population dietary exposures [37,48,49,50,51]. In contrast, direct evidence tracing complete pathways from financialization or food sovereignty to oral-health outcomes remains limited [34,59]. The framework finally contributes by integrating fragmented evidence, distinguishing empirically supported relationships from theoretically plausible pathways, and defining testable propositions for future research.

The structured narrative approach enabled the integration of heterogeneous theoretical, empirical, policy, and mechanistic evidence across disciplines that rarely share common analytical frameworks. However, the absence of exhaustive systematic searching, duplicate screening, formal risk-of-bias assessment, and quantitative synthesis limits conclusions regarding the completeness and comparative strength of the evidence. The interpretive nature of conceptual synthesis also introduces potential subjectivity in selecting and connecting evidence across disciplinary domains. Methodological transparency was strengthened through predefined review questions, explicit analytical domains, structured literature selection, thematic synthesis, and identification of the core sources informing framework development. The proposed framework should therefore be interpreted as an evidence-informed conceptual synthesis rather than a validated causal or predictive model. Overall, this review extends food-system analysis by integrating the distribution of economic power and agency with food environments, dietary exposures, and oral-health outcomes [23,44,45,46]. Its principal contribution is the identification of oral health as a potential biological interface through which structurally patterned dietary exposures and food-system inequalities may be investigated within a One Health perspective [52,53]. The framework thus provides a theoretically grounded basis for testing multilevel relationships among food-system governance, dietary transformation, biological processes, and health inequalities while supporting more integrated approaches to food-system sustainability and One Health research [47,52,62].

## 7. Conclusions

Food sovereignty offers one potential means of moderating these influences by strengthening the agency of citizens, local communities, Indigenous peoples, and food producers in shaping food production and governance. By promoting more equitable and sustainable food systems, it may also help modify the pathways through which financialization influences dietary exposures and, ultimately, health outcomes. A central contribution of this review is the positioning of oral health as a biologically plausible interface linking upstream food-system organization with downstream health outcomes. Because the oral cavity represents the first biological point of contact with the food system, oral-health outcomes may provide complementary evidence of how upstream food-system processes become biologically embodied through dietary exposures and microbial, inflammatory, and metabolic pathways. Rather than representing a validated indicator of food-system performance, oral health offers a complementary and empirically testable dimension through which food-system inequalities and dietary transformations may become observable.

Accordingly, this review extends existing food-security, sustainability, and One Health frameworks by integrating oral health into an interdisciplinary conceptual model linking food-system financialization, governance, food environments, dietary exposures, and health. Although the proposed framework is conceptual rather than causal, it identifies empirically testable pathways that can guide future interdisciplinary research.

Future research should evaluate these pathways across different populations and food-system contexts and determine whether oral-health indicators can complement existing measures of food-system performance, equity, resilience, and sustainability. Particular attention should also be given to the implications of changing global policy priorities, including the reduced emphasis by some international institutions on poverty reduction, climate change, and global health. Understanding how these policy shifts interact with food-system financialization and whether food-sovereignty approaches can reduce their effects represents an important priority for future research and policy development.

## Figures and Tables

**Table 1:** Core references informing the interdisciplinary thematic synthesis and development of the proposed conceptual framework.

Authors, Year [Reference No.]	Study/Source Type	Review Domain	Main Contribution to the Narrative Synthesis	Role in the Conceptual Framework
Clapp, 2020 [15]	Scholarly book	Food-system political economy and financialization	Synthesizes the political, economic, and institutional processes through which markets, financial actors, and power relations shape contemporary food systems.	Provides the principal theoretical foundation for conceptualizing financialization and market power as upstream structural drivers of food-system organization.
Isakson et al., 2023 [16]	Scholarly book chapter	Financialization and food security	Examines how financialization and commodity-market mechanisms affect agricultural systems, producers, and food security.	Supports the pathway linking financial mechanisms and commodity markets with food-system vulnerability and population-level consequences.
Clapp, 2018a [56]	Conceptual article	Financialization and global food politics	Explains how financialization increases the distance between financial decision-making, food production, and populations affected by food-system outcomes.	Establishes financialization as an upstream structural process influencing power relations, food-system organization, and downstream exposures.
Clapp, 2018b [57]	Conceptual article	Corporate concentration and market power	Examines corporate consolidation, mega-mergers, market concentration, and their implications for power and sustainability in global food systems.	Identifies corporate concentration as a key intermediate mechanism connecting financialization with market power and food-system governance.
Clapp et al., 2022 [23]	Conceptual framework article	Food security, agency, and sustainability	Extends the traditional four-dimensional food-security model by incorporating agency and sustainability as essential dimensions.	Provides the principal food-security architecture for integrating agency, governance, sustainability, and power into the proposed framework.
Stuckler and Nestle, 2012 [58]	Conceptual article	Commercial determinants of health	Examines how transnational food corporations and commercial practices influence food environments, dietary patterns, and population health.	Supports the pathway linking corporate power and commercial practices with population dietary exposures and downstream health outcomes.
Borras and Mohamed, 2020 [59]	Conceptual article	Food sovereignty and health equity	Connects food sovereignty and food security with structural inequalities, political economy, and health inequities.	Supports the integration of food sovereignty, power distribution, equity, and population health within the governance dimension of the framework.
Wittman, 2015 [60]	Conceptual and policy article	Food sovereignty and governance	Examines the challenges involved in translating food-sovereignty principles from social movements into institutional policy and governance arrangements.	Supports the institutional and governance mechanisms through which food sovereignty may modify food-system structures and outcomes.
Sampson et al., 2021 [61]	Systematic review	Food sovereignty, food security, and nutrition	Synthesizes evidence concerning food sovereignty and rights-based approaches in relation to food security and nutritional outcomes.	Provides empirical support for food sovereignty as a governance pathway linking agency and community control with nutrition, equity, and food security.
Papargyropoulou et al., 2025 [62]	Research framework article	Food security and sustainability	Develops an interdisciplinary framework for examining food security and sustainability across interconnected food-system dimensions and scales.	Provides a contemporary framework against which the proposed model is positioned and differentiated, particularly regarding integration of biological and oral-health outcomes.
HLPE, 2020 [54]	International expert report	Food security, nutrition, agency, and sustainability	Provides an integrated framework for understanding food security and nutrition through the dimensions of availability, access, utilization, stability, agency, and sustainability.	Provides the multidimensional food-security architecture underlying the proposed framework and its governance, agency, and sustainability dimensions.
Mackenbach et al., 2022 [44]	Systematic review	Food environments and oral health	Synthesizes evidence concerning relationships between food-environment characteristics, dietary exposures, oral-health outcomes, and inequalities.	Provides a central empirical bridge connecting upstream food environments with downstream oral-health outcomes and inequalities.
Curca et al., 2026 [45]	Narrative review	Nutrition and oral health	Synthesizes dietary and biological pathways through which nutritional exposures influence oral and periodontal health.	Supports the mechanistic pathway connecting population dietary exposures with biological processes and oral-health outcomes.
de Abreu et al., 2021 [46]	Conceptual article	Structural determinants of oral health	Positions oral health within broader social, environmental, and structural determinants and emphasizes the unequal distribution of oral diseases.	Supports the interpretation of oral-health inequalities as biologically embodied consequences of structurally patterned food environments and exposures.
Moussa et al., 2025 [47]	Conceptual article	Oral health and One Health	Examines the relevance and potential contribution of oral health to One Health research, policy, and interdisciplinary collaboration.	Provides the principal conceptual basis for integrating oral health into the One Health dimension of the proposed framework.
Wood et al., 2023 [34]	Exploratory analysis	Financialization and ultra-processed foods	Examines financialization within the ultra-processed food industry and its influence on corporate strategies, market expansion, and public-health outcomes.	Provides the critical analytical bridge linking financialization, shareholder-oriented corporate strategies, UPF expansion, dietary exposures, and health outcomes.
Baker and Friel, 2016 [36]	Review	Food-system transformation and nutrition transition	Examines structural transformations in food systems and the political-economic drivers contributing to changing food environments and dietary patterns.	Supports the pathway connecting food-system restructuring, globalization, and corporate expansion with changing food environments and population dietary exposures.
Baker et al., 2020 [37]	Synthesis review	Political economy of ultra-processed foods	Synthesizes global trends and political-economic drivers underlying the production, distribution, marketing, and consumption of ultra-processed foods.	Supports the intermediate pathway connecting structural and commercial food-system drivers with UPF expansion and population dietary exposures.
Moodie et al., 2021 [43]	Synthesis review	Corporate practices and ultra-processed foods	Examines market and political practices through which transnational corporations promote and sustain the global expansion of ultra-processed foods.	Supports the commercial mechanisms linking corporate power, market concentration, food environments, and population dietary exposures.
Dimopoulou et al., 2023 [48]	Narrative review	Nutrition and dental caries	Synthesizes evidence on nutritional exposures and biological mechanisms associated with dental caries across the life course.	Supports the dietary and biological pathways connecting food-system-related exposures with oral disease development.
Cascaes et al., 2023 [50]	Systematic review and meta-analysis	Ultra-processed foods and dental caries	Synthesizes empirical evidence concerning associations between ultra-processed food consumption and dental caries among children and adolescents.	Provides direct empirical support for the pathway linking structurally shaped dietary exposures and UPF consumption with measurable oral-health outcomes.
WHO, 2023 [52]	International framework	One Health	Defines the contemporary One Health approach and emphasizes the interdependence of human, animal, and ecosystem health within integrated systems.	Provides the overarching One Health architecture within which food systems, environmental conditions, biological pathways, and oral health are integrated.
Baroni de Carvalho et al., 2023 [53]	Conceptual article	One Health and oral health	Examines the relevance of the One Health initiative to oral health and highlights opportunities for interdisciplinary integration.	Supports the conceptual inclusion of oral health within One Health research and the positioning of oral health as a component of integrated health frameworks.

## Data Availability

No new data were created or analyzed in this study. Data sharing is not applicable to this article.
